# *Wolbachia* infection-responsive immune genes suppress *Plasmodium falciparum* infection in *Anopheles stephensi*

**DOI:** 10.1371/journal.ppat.1012145

**Published:** 2024-04-10

**Authors:** Vandana Vandana, Shengzhang Dong, Tanaya Sheth, Qiang Sun, Han Wen, Amanda Maldonado, Zhiyong Xi, George Dimopoulos

**Affiliations:** 1 W. Harry Feinstone Department of Molecular Microbiology and Immunology, Bloomberg School of Public Health, Johns Hopkins University, Baltimore, Maryland, United States of America; 2 Department of Microbiology and Molecular Genetics, Michigan State University, East Lansing, Michigan, United States of America; National Institutes of Health, UNITED STATES

## Abstract

*Wolbachia*, a maternally transmitted symbiotic bacterium of insects, can suppress a variety of human pathogens in mosquitoes, including malaria-causing *Plasmodium* in the *Anopheles* vector. However, the mechanistic basis of *Wolbachia*-mediated *Plasmodium* suppression in mosquitoes is not well understood. In this study, we compared the midgut and carcass transcriptomes of stably infected *Anopheles stephensi* with *Wolbachia w*AlbB to uninfected mosquitoes in order to discover *Wolbachia* infection-responsive immune genes that may play a role in *Wolbachia*-mediated anti-*Plasmodium* activity. We show that *w*AlbB infection upregulates 10 putative immune genes and downregulates 14 in midguts, while it upregulates 31 putative immune genes and downregulates 15 in carcasses at 24 h after blood-fed feeding, the time at which the *Plasmodium* ookinetes are traversing the midgut tissue. Only a few of these regulated immune genes were also significantly differentially expressed between *Wolbachia*-infected and non-infected midguts and carcasses of sugar-fed mosquitoes. Silencing of the *Wolbachia* infection-responsive immune genes *TEP 4*, *TEP 15*, *lysozyme C2*, *CLIPB2*, *CLIPB4*, *PGRP-LD* and two novel genes (a *peritrophin-44-like* gene and a *macro domain-encoding gene*) resulted in a significantly greater permissiveness to *P*. *falciparum* infection. These results indicate that *Wolbachia* infection modulates mosquito immunity and other processes that are likely to decrease *Anopheles* permissiveness to *Plasmodium* infection.

## Author summary

*Wolbachia* is a maternally transmitted symbiotic bacterium of many insects, and in certain vector mosquitoes it can suppress human pathogens including dengue virus in *Aedes* mosquitoes and malaria-causing *Plasmodium* in the *Anopheles* vector. The mechanism by which *Wolbachia* infection of the mosquito suppresses the *Plasmodium* parasite is not well understood. In this study, we compared expression of *Anopheles stephensi* genes with and without *Wolbachia* infection to discover *Wolbachia* infection-responsive immune genes that may be responsible for *Plasmodium* suppression as part of the mosquitoes anti-*Plasmodium* immune response. We show that *Wolbachia* infection of the *Anopheles stephensi* malaria vector mosquito will regulate numerous putative mosquito immune genes the time at which the *Plasmodium* ookinetes are traversing the midgut tissue after ingestion of an infectious blood meal. We also show through functional studies that several of the *Wolbachia* infection-responsive immune genes *TEP 4*, *TEP 15*, *lysozyme C2*, *CLIPB2*, *CLIPB4*, *PGRP-LD* and two novel genes (a *peritrophin-44-like gene* and a *macro domain-encoding gene*) are indeed involved in suppressing *P*. *falciparum* infection. Our study show that *Wolbachia* infection modulates mosquito immunity and other processes that are likely to impact *Anopheles* ability to transmit malaria.

## Introduction

*Wolbachia*, a maternally transmitted symbiotic bacterium of certain mosquito species, has already been developed as a tool for the control of arboviral diseases such as dengue. *Wolbachia* has been shown to modify *Aedes aegypti* mosquito biology in ways that make the mosquitoes less permissive to arboviruses, rendering the mosquitoes incapable of transmitting these diseases [1–3]. Given the previous success in using *Wolbachia* as a virus transmission-blocking strategy in *Ae*. *aegypti*, efforts are now being made to expand this strategy to anopheline mosquitoes that are vectors of malaria [4]. Previous studies involving transient somatic infection have indicated that *Wolbachia* may impair *Plasmodium* transmission in *Anopheles* mosquitoes, possibly by inducing mosquito antiparasitic immune responses [5,6]. These *Wolbachia*-related effects support the feasibility of *Wolbachia*-based interventions for malaria vector control, but only if stable *Wolbachia* infections can be established in *Anopheles* malaria vectors.

While early attempts to establish stable *Wolbachia* infection in *anopheline* mosquitoes were unsuccessful, several recent studies have detected *Wolbachia* DNA in various *Anopheles species* [7–14]. Bian et al. (2013) generated the first stable *Wolbachia* (wAlbB)-infected *An*. *stephensi* LB1 line [15], leading to successful maternal transmission and cytoplasmic incompatibility (CI). Importantly, the *wAlbB*-transinfected *An*. *stephensi* showed significantly reduced permissiveness to *P*. *falciparum* and *P*. *berghei* when compared to non-*Wolbachia*-infected control mosquitoes [15,16]. A study by Chen et al. [17] showed that the microbiome of *An*. *stephensi* remains unaffected upon *Wolbachia* infection, suggesting that *Wolbachia*-mediated *Plasmodium* suppression does not involve the mosquito microbiome. Two other studies, by Kambris et al. [6] and Hughes et al. [5], have indicated that laboratory-reared *An*. *gambiae* that are transiently infected with wMelPop and wAlbB become resistant to *P*. *berghei* and *P*. *falciparum* infection. However, several studies have shown that the effect of *Wolbachia* on *Plasmodium* infection in *Anopheles* can vary depending on the *Wolbachia* strain and the parasite and mosquito species involved. For example, two studies have shown that natural *Wolbachia* infection in *An*. *coluzzii* and *An*. *gambiae* field mosquitoes is negatively correlated with *Plasmodium* development [11,18]. Other studies have indicated that the *Wolbachia* wPip strain renders *Culex pipiens* mosquitoes more permissive to the avian malaria parasite *P*. *relictum* [19] and that *Wolbachia* infection does not influence *P*.* falciparum* development in *An*.* moucheti* mosquitoes [20].

The molecular mechanisms underlying *Wolbachia*-mediated anti-*Plasmodium* activity in mosquitoes are not completely understood. Bian et al. (2013) have shown that *wAlbB*-induced reactive oxygen species (ROS) potentially play a role in the mosquitoes’ resistance to *Plasmodium* infection [15]. Similarly, *Wolbachia* induced ROS-dependent activation of the Toll pathway is associated with anti-viral protection in *Ae*. *aegypti* [21]. Furthermore, Joshi et al. (2017) have shown that mosquito immune genes such as defensin, *TEP1*, *PGRP* and *LRMs* are upregulated upon *Wolbachia* infection and are possibly involved in regulation of *P*. *berghei* infection in *wAlbB*-infected mosquitoes [16].

To examine the role of the mosquito’s immune response in *Wolbachia*-mediated anti-*Plasmodium* activity, we have now analyzed the midgut and carcass transcriptomes of sugar-fed versus blood-fed *An*. *stephensi* that are stably infected with *Wolbachia* (*wAlbB*). Focusing on the immune genes that were significantly regulated by *Wolbachia* infection, we investigated a potential role for selected upregulated genes in modulating *Plasmodium* infection in *An*. *stephensi*. We demonstrated that silencing of *Wolbachia*-regulated *TEP 4*, *TEP 15*, *lysozyme C2*, and *CLIPB2*, *CLIPB4*, *PGRP-LD*, *peritrophin-44 like*, and *macrodomain* genes leads to significant increases in *P*. *falciparum* infection intensity in *An*. *stephensi* mosquitoes, indicating that these genes may suppress *Plasmodium* infection in *Wolbachia*-infected *Anopheles*.

## Results

### Changes in the midgut and carcass transcriptomes of *An*. *stephensi in response to Wolbachia* infection

**Fig 1:**
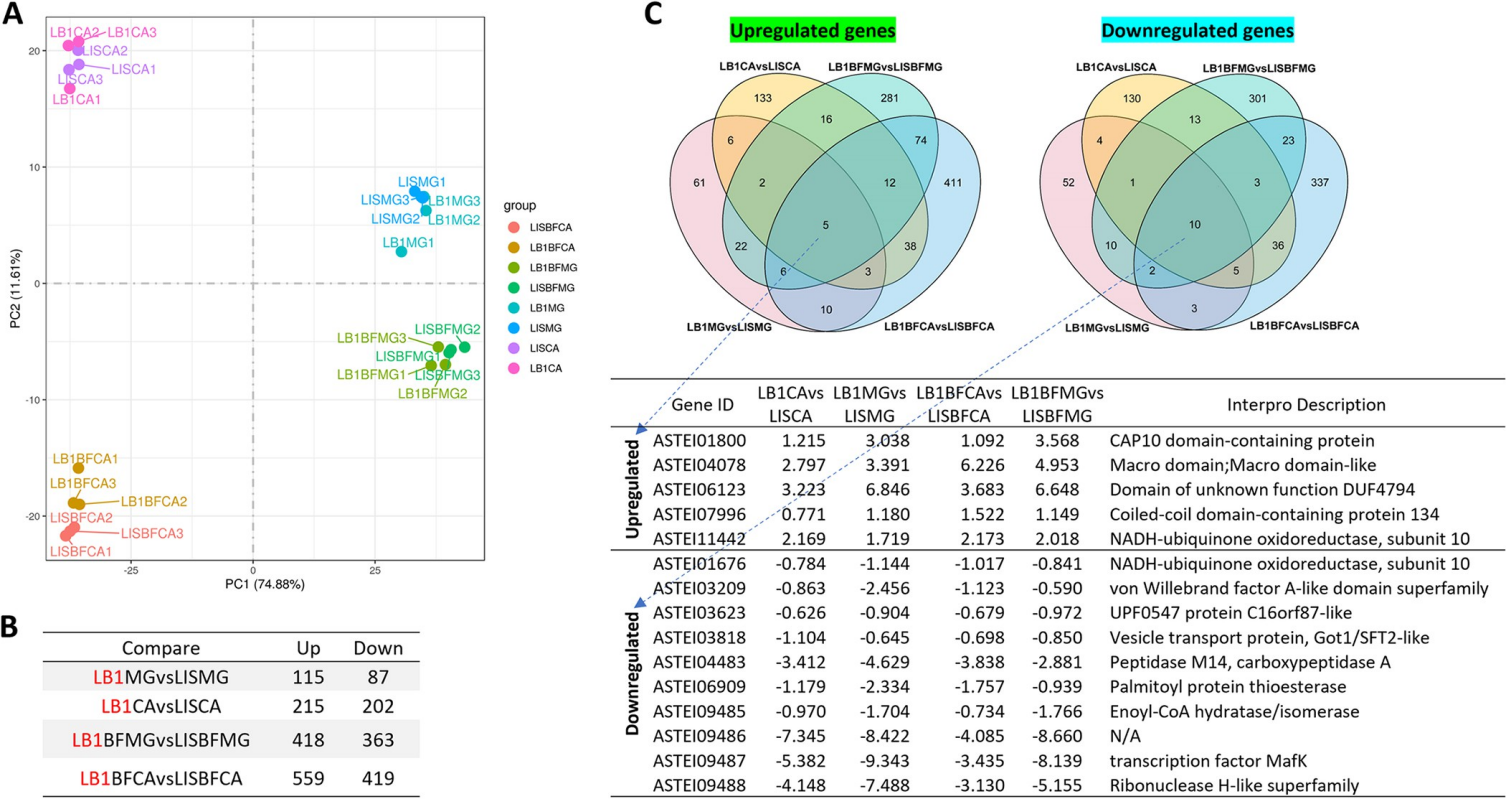
Transcriptome analysis of *Wolbachia*-regulated genes in midguts (MG) and carcasses (CA) of *An*. *stephensi* at day 1 post-blood feeding (blood-fed, BF) or without blood feeding (sugar-fed). Each sample had three biological replicates. (**A**) PCA analysis of midgut and carcass transcriptomes. (**B**) Number of upregulated and downregulated differentially expressed (DE) genes in midguts and carcasses of *Wolbachia*-infected (LB1) mosquitoes as compared to those of uninfected (LIS) mosquitoes. (**C**) Shared upregulated or downregulated DE genes in midguts and carcasses of blood-fed and sugar-fed LB1. Table shows log2 -fold change and description of the shared *Wolbachia*-regulated genes. LB1MG: *Wolbachia*-infected, sugar-fed midguts; LB1CA: *Wolbachia*-infected, sugar-fed carcasses; LISMG: uninfected, sugar-fed midguts; LISCA: uninfected, sugar-fed carcasses; LB1BFMG: *Wolbachia*-infected, blood-fed midguts; LB1BFCA: *Wolbachia*-infected, blood-fed carcasses; LISBFMG: uninfected, blood-fed midguts; LISBFCA: uninfected, blood-fed carcasses.

To determine the effect of *Wolbachia* infection on gene expression in the midguts and carcasses (the mosquito whole body without the midgut) of *An*. *stephensi*, we compared midgut and carcass transcriptomes between *Wolbachia*-infected (LB1) and uninfected (LIS) females at 1-day post-blood feeding (blood-fed) or without blood feeding (sugar-fed). Overall, ~90% of the sequencing data could be aligned with the *An*. *stephensi* genome (S1 Table). Principal component analysis (PCA) showed four distinct clusters for the sugar-fed midguts, blood-fed midguts, sugar-fed carcasses, and blood-fed carcasses (Fig 1A). In each cluster, three *Wolbachia*-infected or uninfected samples were associated with each other, except in the cluster of sugar-fed carcasses, in which LISCA2, LB1CA2, and LB1CA3 transcriptomes were closely related (Fig 1A). Hundreds of genes were significantly upregulated or downregulated in response to *Wolbachia* infection in midguts and carcasses, with and without blood feeding (Fig 1B), but the number of *Wolbachia*-regulated genes was higher in blood-fed mosquitoes than in sugar-fed mosquitoes. Five and ten shared genes were significantly upregulated and downregulated, respectively, in *Wolbachia*-infected samples as compared to their corresponding uninfected samples (Fig 1C).

### Enrichment of Immune genes in the carcasses of *Wolbachia*-infected *An*. *stephensi*

**Fig 2:**
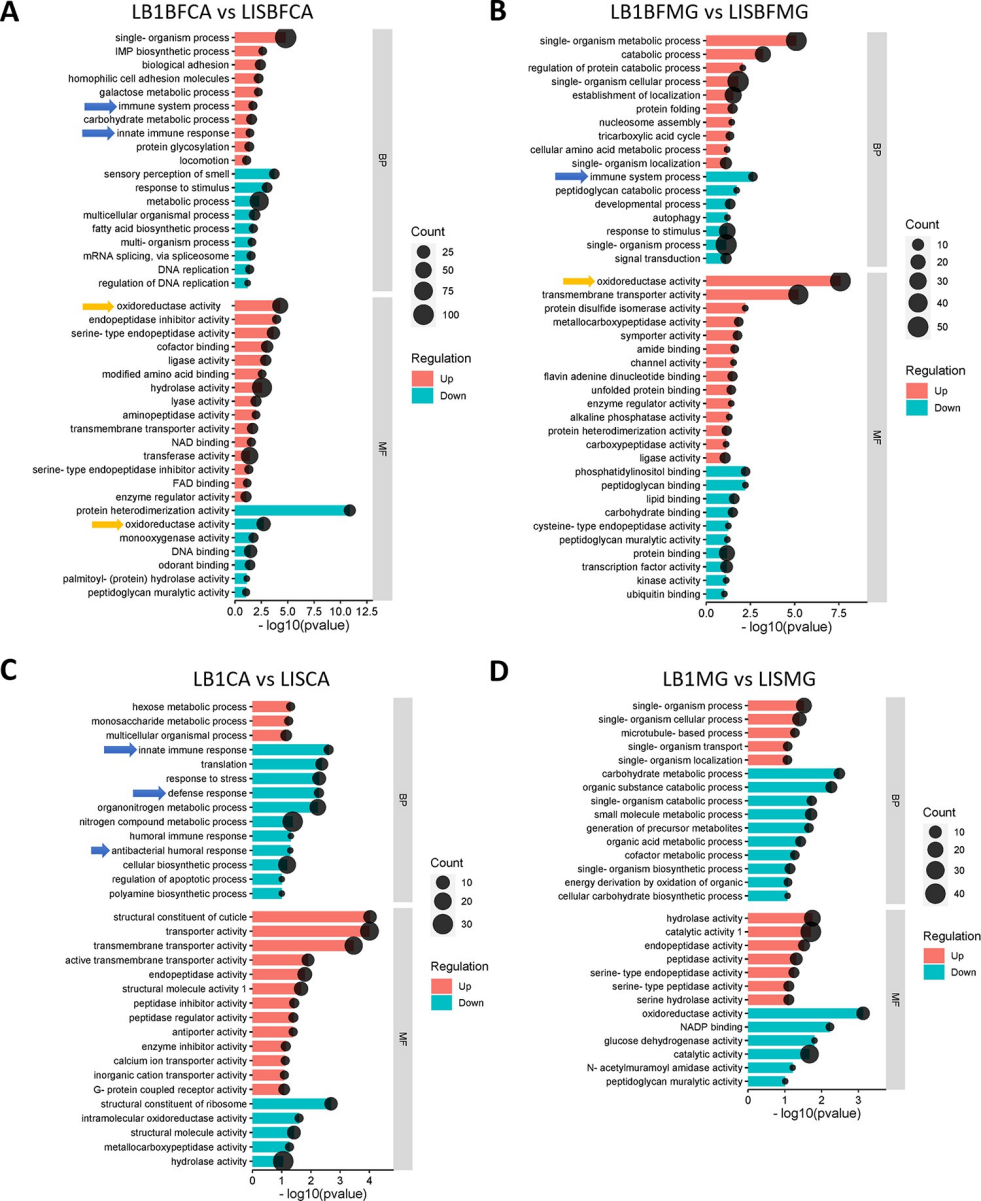
Gene ontology (GO) analysis of *Wolbachia*-regulated genes in midguts (MG) and carcasses (CA) of blood-fed (A and B) and sugar-fed (C and D) *An*. *stephensi*. LB1, *Wolbachia*-infected; LIS, uninfected; BP, biological process; MF, molecular function. Blue arrows indicate genes with immune/defense function. Orange arrows indicate genes with oxidoreductase activity.

**Fig 3:**
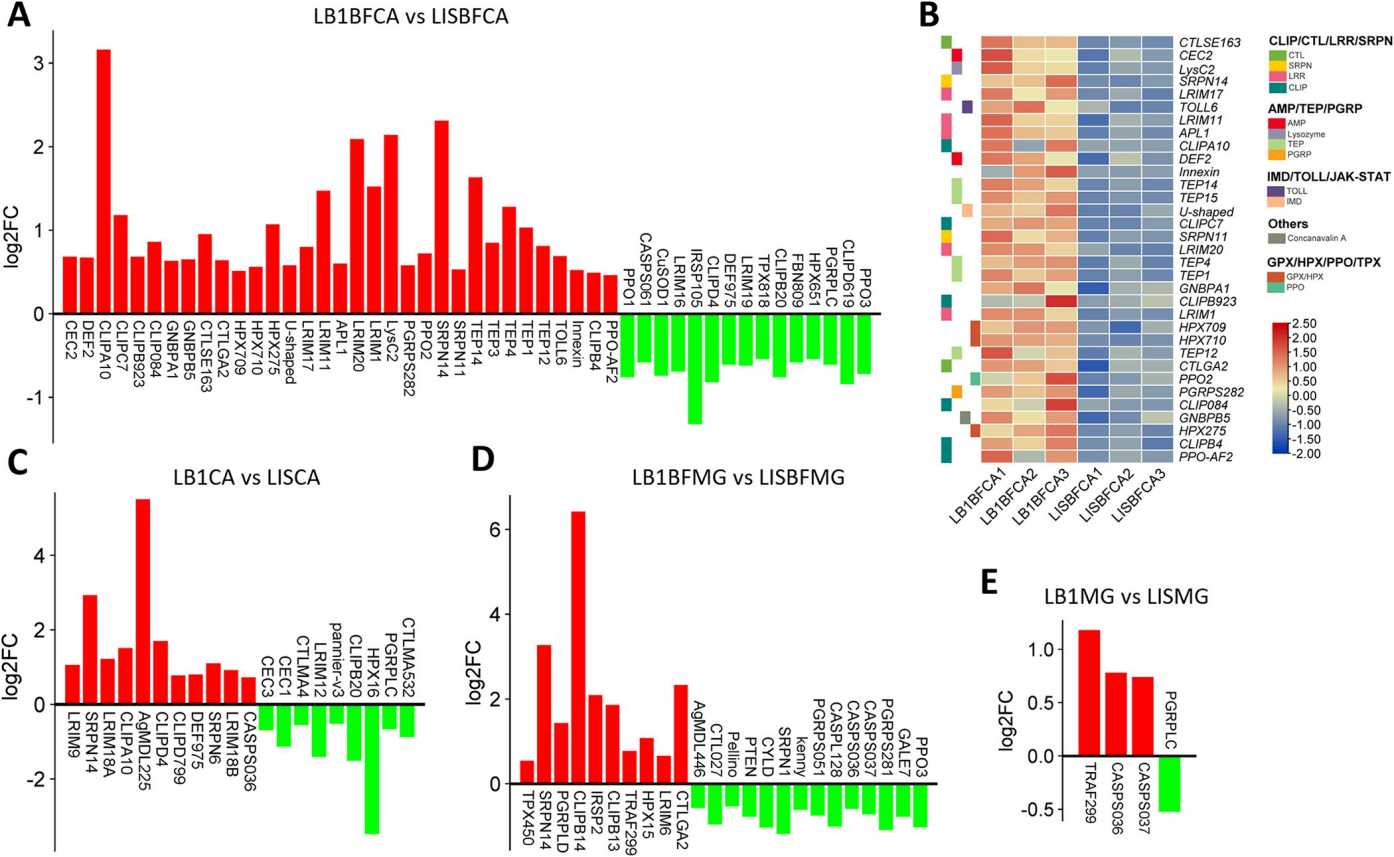
Transcriptome analysis of *Wolbachia*-regulated immune genes in midguts (MG) and carcasses (CA) of *An*. *stephensi*. (**A)** -Fold change (FC) of differentially expressed (DE) immune genes in carcasses of blood-fed (BF) *Wolbachia*-infected (LB1) mosquitoes as compared to uninfected (LIS) carcasses. (**B)** Heatmap showing expression of *Wolbachia*-upregulated immune genes classified into different groups in carcasses of blood-fed *Wolbachia*-infected mosquitoes. Three biological replicates were included in the heatmap. Fold change (log2) of expression of DE immune genes in carcasses of sugar-fed (**C**) and midguts of blood-fed (**D**) and sugar-fed (E) *Wolbachia*-infected mosquitoes as compared to those of uninfected mosquitoes.

GO analyses of upregulated and downregulated DE genes showed that innate immune response process and defense response were among the significantly regulated biological process (BP) and molecular function (MF) groups that also included metabolic process, single-organism process, hydrolase activity, and lipid binding groups (Fig 2). These predicted immune and defense response genes include genes involved in innate immune responses, immune system processes, defense responses, antibacterial humoral responses, and oxidoreductase, suggesting *Wolbachia* infection may modulate the immune and stress responses in mosquitoes. Previous studies have shown upregulation of six immune genes, *TEP1*, *REL1*, *PGRPLC*, *DEF1*, *LRM1*, and *CAT1*, in *Wolbachia*-infected midguts and carcass tissues [16]. Consistently, in carcasses of blood-fed mosquitoes, we found that *Wolbachia* infection led to 31 upregulated and 15 downregulated immune genes (Fig 3A and S2 Table), and the upregulated genes included predicted *AMP*, *leucin-rich repeats (LRMs)*, *defensin*, *lysozyme*, *C-type lectin-like (CTL)*, and *CLIP* domain serine protease and *TEP* genes (Fig 3B and S2 Table). In carcasses of sugar-fed mosquitoes, 11 and 9 immune genes were upregulated and downregulated, respectively, in *Wolbachia*-infected mosquitoes as compared to uninfected mosquitoes, and also among the many upregulated immune genes were *CTL* and *CLIP-domain serine protease* genes; among the downregulated genes were three AMP genes (Fig 3C). These data suggest that *Wolbachia* infection induces a more prominent immune response in blood-fed mosquitoes than in sugar-fed mosquitoes.

We also compared the differential expression of immune genes in the midguts of *Wolbachia*-infected and uninfected blood-fed and sugar-fed mosquitoes. In the midguts of blood-fed mosquitoes, 10 and 14 immune genes were upregulated and downregulated, respectively, by the presence of *Wolbachia*, and these genes had diverse functions (Fig 3D). However, *Wolbachia* infection led to the upregulation of only three genes and downregulation of only one gene in the midguts of sugar-fed mosquitoes (Fig 3E). Therefore, transcriptome analysis of both the carcasses and midguts supports the conclusion that *Wolbachia*-induced immune responses mainly occur in mosquitoes after ingestion of a blood meal, likely as a result of the increase in *Wolbachia* density that occurs upon blood feeding [22]. Our analysis also indicates that *Wolbachia* infection stimulates a weaker immune response in midguts than in carcasses.

### Testing selected *Wolbachia*-regulated immune genes for anti-*Plasmodium* activity

Since stable *Wolbachia* (wAlbB) infection can suppress *Plasmodium* in *An*. *stephensi* and modulate expression of many immune genes, we hypothesized that at least some of these immune genes are likely to play a role in regulating *Plasmodium* infection in mosquitoes. To test this hypothesis, we selected 18 genes that were significantly regulated by *Wolbachia* infection (Table 1). Among these genes, three upregulated genes encoding the *CAP10 domain* (ASTEI01800), *macrodomain* (ASTEI04078), and DUF4794 domain (ASTEI06123) and three downregulated genes encoding *carboxypeptidase A* (ASTEI04483), *peritrophin-44 like* (ASTEI09413), and *transcription factor MafK* (ASTEI09487) were of unknown function with regard to immunity and anti-*Plasmodium* defense (Fig 1C). In addition, we selected 4 putative immune genes predicted to encode *C-type lectin like (*ASTEI10532) [23], *3- glucan binding protein (*ASTEI08630) [24], *cecropin C2 (*ASTEI01170) [25], *PGRPLD* (ASTEI02158) [26], based on their role in anti-*Plasmodium* defense as reported previously. Moreover, 8 genes namely, predicted *lysozyme C2* (ASTEI01308), *CLIPB2* (ASTEI08923), *CLIPB4 (*ASTEI08922), *CLIP-like (*ASTEI02516), *PPO-activating factor 2 (*ASTEI04157), *TEP 4 (*ASTEI08428), *TEP 14* (ASTEI06643) *and TEP 15 (*ASTEI06644), were found significantly upregulated in *Wolbachia*-infected blood-fed mosquitoes and were selected to examine their role in anti-*Plasmodium* activity (Table 1).

**Table 1:** *Wolbachia*-regulated genes selected for their role in anti-*Plasmodium* activity.

Gene ID	Gene Description (based on ortholog prediction from *An*. *gambiae* and phylogenetic analysis)	Selection criteria
ASTEI01800	CAP10 domain containing protein	Shared upregulated gene
ASTEI06123	DUF4794 domain containing protein	Shared upregulated gene
ASTEI04078	Macrodomain containing protein	Shared upregulated gene
ASTEI09487	Transcription factor MafK	Shared downregulated gene
ASTEI04483	Carboxypeptidase A	Shared downregulated gene
ASTEI09413	Peritrophin-44 like	Shared downregulated gene
ASTEI10532	C-type lectin-like (CTLMA532)	Anti-*Plasmodium* gene
ASTEI02158	Peptidoglycan recognition protein long D (PGRP-LD)	Anti-*Plasmodium* gene
ASTEI08630	3-glucan binding protein (GNBPA1)	Anti-*Plasmodium* gene
ASTEI01170	Cecropin C2 (CEC2)	Anti-*Plasmodium* gene
ASTEI08923	CLIPB2	Putative immune gene
ASTEI01308	Lysozyme C2 (LysC2)	Putative immune gene
ASTEI08922	CLIPB4	Putative immune gene
ASTEI02516	CLIP-like	Putative immune gene
ASTEI04157	PPO-activating factor 2 like (PPO-AF2)	Putative immune gene
ASTEI08428	TEP4	Putative immune gene
ASTEI06644	TEP15	Putative immune gene
ASTEI06643	TEP14	Putative immune gene

**Fig 4:**
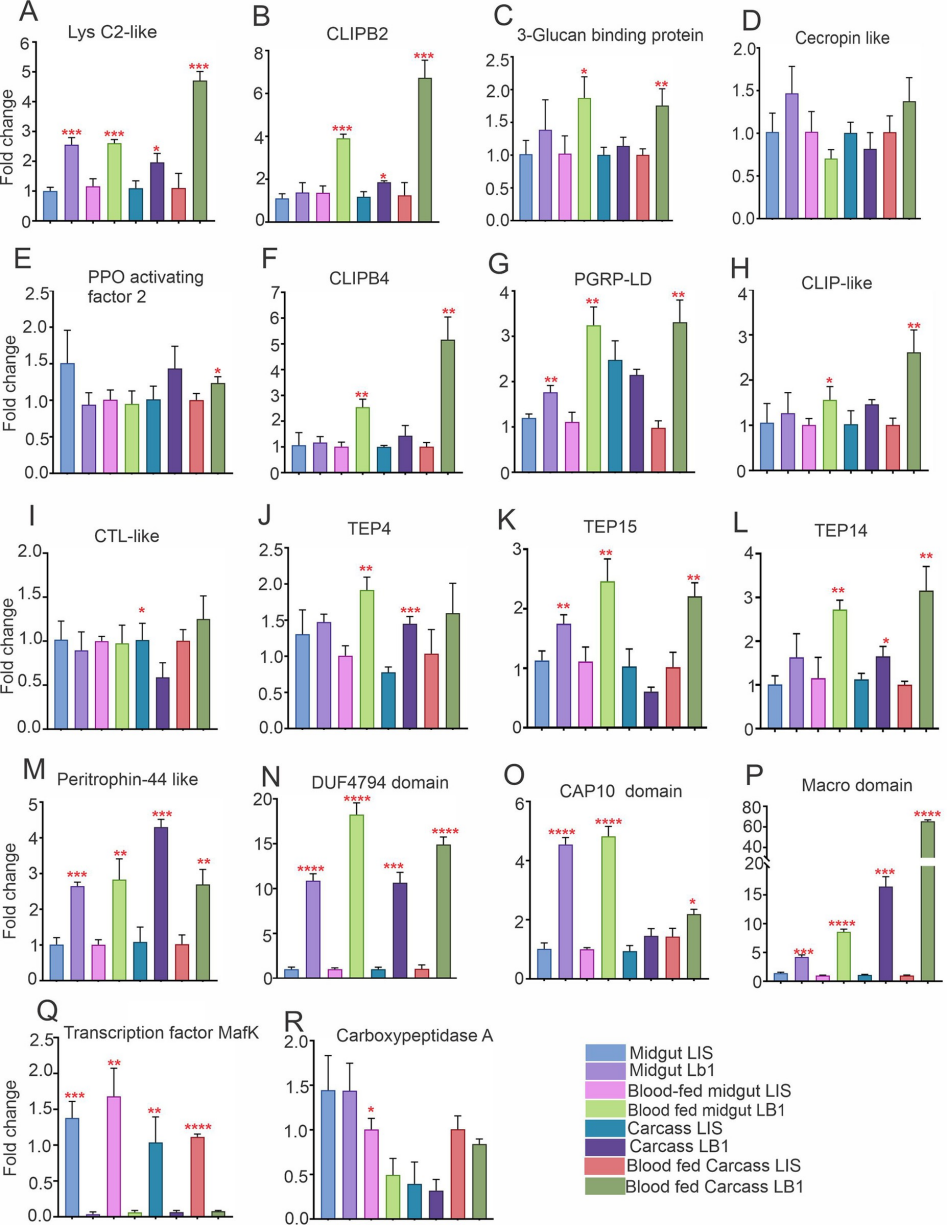
Tissue-specific expression of 18 selected immune genes in the *Wolbachia*-infected *An*. *stephens*i. Gene expression was analyzed using qPCR in the midguts and carcasses of *Wolbachia*-infected (LB1) and uninfected (LIS) mosquitoes. The genes with increased expression in *Wolbachia*-infected midgut and carcasses tissues by qPCR, were depicted to be upregulated in the RNA seq experiments. Error bars indicate the SEM of three biological replicates, each containing 5–10 adult females. Statistical significance was determined using the Mann-Whitney test between *Wolbachia*-infected tissue versus uninfected tissues samples. * *P* < 0.05; ** *P* < 0.01; ***P < 0.001; **** *P* <0.0001.

We used qPCR to validate the differential expression of the selected genes in midgut and carcass tissues of the wAlbB-infected and uninfected *An*. *stephensi*. Among the selected genes, *lysC2*, *CLIPB2*, *3-glucan binding protein*, *CLIPB4*, *PGRP-LD*, *CLIP-like*, *TEP4*, *TEP15*, and *TEP14* showed significantly higher expression in both blood-fed midguts and carcasses of the wAlbB-infected (LB1) mosquitoes than of the uninfected (LIS) *An*. *stephensi* (Fig 4A–4L). Of these genes, *CLIPs*, *TEPs*, *PGRP-LD*, and *PPO* have previously been linked to inhibitory or antagonistic effects on *Plasmodium* development [27–31]. Some novel genes, namely, *peritrophin-44 like*, *DUF4764 domain-containing*, *CAP10 domain-containing*, *Macro domain-containing*, and *transcription factor MafK*, also showed significantly higher expression in both the midguts and carcasses of the wAIbB-infected mosquitoes than in uninfected *An*. *stephensi* (Fig 4M–4R). However, the role of these genes in anti-*Plasmodium* activity has not previously been examined.

**Fig 5:**
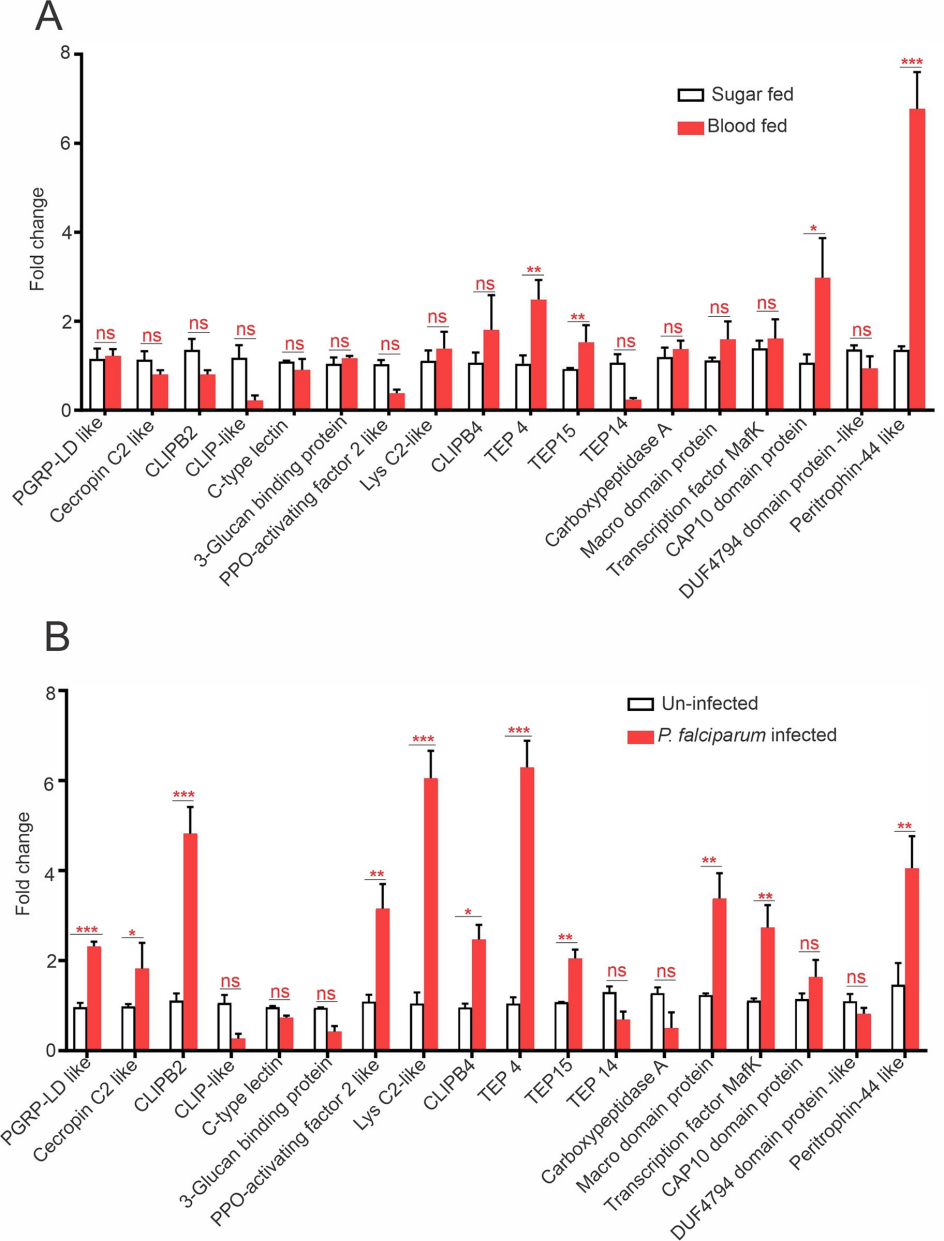
Expression of 18 selected immune genes in blood-fed and *P*. *falciparum* infected *Wolbachia-* free *An*. *stephens*i *LIS* strain. **(A)** Gene expression was analyzed by qPCR. Gene expression of blood-fed versus sugar-fed mosquitoes. Predicted *TEP4*, *TEP15* and *CAP10 domain protein* gene expression increased in blood-fed samples whose expression was found upregulated in *Wolbachia*-infected midgut and carcass tissues as shown in figure **(B)** Gene expression in *P*. *falciparum*-infected versus uninfected female mosquitoes. Error bars indicate the SEM of three biological replicates, each containing 5–10 adult females. Statistical significance was determined using the Mann-Whitney test between sugar fed versus blood fed samples (A) and *P*. *falciparum* uninfected versus infected samples (B); * *P* < 0.04; ** *P* < 0.02; *** *P* < 0.002.

We also assessed the differential mRNA abundance of selected genes in blood-fed versus sugar-fed, and in *P*. *falciparum*-infected versus uninfected laboratory-reared *Wolbachia* uninfected *An*. *stephensi* mosquitoes. Among the 18 selected genes, *TEP 4*, *TEP 15*, *CAP10 domain-containing protein*, and *peritrophin-44-like* showed significantly increased expression in blood-fed females as compared to sugar-fed females (Fig 5A). In addition to *TEP 4*, *TEP 15*, and *peritrophin-44 like*, the expression of *CLIPB2*, *CLIPB4*, *PGRP-LD*, *lysC2*, *PPO-activating factor 2*, *cecropin C2*, *macro domain protein* and *transcription factor MafK* was significantly increased in *P*. *falciparum*-infected mosquitoes as compared to uninfected mosquitoes (Fig 5B).

### Increase in *P*. *falciparum* infection after silencing of the selected *Wolbachia*-regulated genes in *An*. *stephensi*

**Fig 6:**
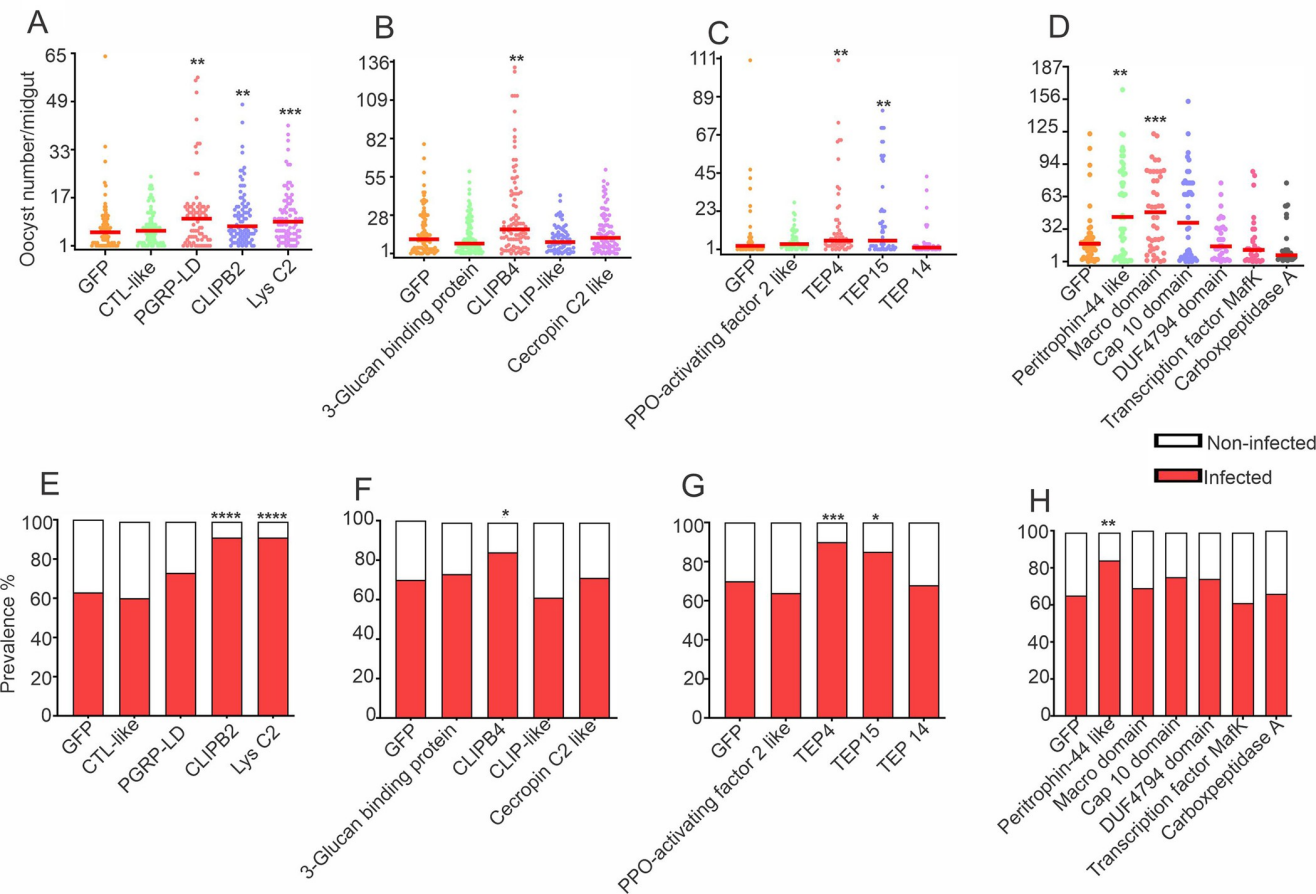
Effect of silencing of the 18 selected immune genes on *Plasmodium falciparum* infection in *An*. *stephensi*. Female mosquitoes were injected with dsRNA of each of the immune genes; dsRNA of GFP was used as a control. (**A-D**) *P*. *falciparum* intensity in individual midguts of immune gene-silenced mosquitoes. Each dot indicates the number of oocysts in the individual midgut, and horizontal red bars indicate the median value. **(E-H**) *P*. *falciparum* infection prevalence in immune gene-silenced mosquitoes. Two-tailed *P* values obtained with the Mann-Whitney test (infection intensity) or Fisher exact test (infection prevalence) are shown: *, *P* < 0.05; **, *P* < 0.01; ***, *P* < 0.001.

To determine whether a *wAlb*B infection-response gene can influence mosquito permissiveness to *P*. *falciparum* infection, we investigated the effect of gene silencing on *P*. *falciparum* infection in non-*Wolbachia*-infected *An*. *stephensi*. The silencing efficiency of the selected genes is shown in S1 Fig. RNAi-mediated silencing of *PGRP-LD*, *CLIP-B2*, *lysC2* (Fig 6A), and *CLIPB4* (Fig 6B) resulted in a significantly (p<0.001) higher intensity of infection with *P*. *falciparum* (as measured by oocyst numbers in the midgut at 7 days post-feeding on *Plasmodium* gametocytes) as compared to the GFP control. Similarly, knockdown of two *TEP* genes, *TEP4* and *TEP15*, also resulted in a significantly higher oocyst load, indicating a likely role for these genes in *Wolbachia*-mediated *Plasmodium* suppression (Fig 6C). Furthermore, significantly increased oocyst counts were observed upon silencing the peritrophin-44-like and macro domain genes (Fig 6D). Notably, the infection prevalence was significantly increased in *CLIPB2*, *lysC2*, *CLIPB4*, *TEP4*, *TEP15*, and *perotrophin-44 like* gene-silenced mosquitoes, but not significantly in *PGRP-LD-* and macro domain-silenced mosquitoes (Fig 6E–6H).

In summary, silencing of 8 of the 18 selected *Wolbachia*-regulated genes significantly increased the *P*. *falciparum* infection intensity in *An*. *stephensi*. Our results, for the first time, provide evidence for a significant contribution of *Wolbachia*-induced upregulation of the *CLIPs*, *TEPs*, *lysC2*, *PGRP-LD*, *peritrophin-44 like* and *macro domain* genes to suppress *Plasmodium* infection and development in *Anopheles*.

### Orthologs of selected *Wolbachia*-regulated *An*. *stephensi* genes

To gain insight into the possible orthologs and function of the selected *Wolbachia*-induced *An*. *stephensi* genes, we performed a phylogenetic analysis using the protein sequence of the putative orthologs from *An*. *gambiae* or other insect species. The sequence alignment and phylogenetic analysis of three predicted *CLIPs* gene showed that two (ASTEI08922 and ASTEI08923) were clustered together with the *An*. *gambiae CLIPB4* and *CLIPB2* branches, respectively, as their putative orthologs (S2A and S2B Fig). The same pattern was not observed with the third predicted CLIP gene (ASTI02516), indicating that this gene did not belong to *CLIPs* family and explaining the difference in infection phenotype of gene-silenced *An*. *stephensi* after *P*. *falciparum* infection (S2A and S2B Fig). Similarly, phylogenetic analysis of predicted *PGRP (*ASTEI02158), *lysozyme C2* (ASTEI01308), and three *TEPs* genes (ASTEI08428, ASTEI06643, and ASTEI06644) indicated that these genes correspond to *An*. *gambiae PGRP-LD* (S2C Fig), *lysC2* (S3A and S3B Fig), and *TEP4*, *TEP14*, and *TEP15* (S4A and S4B Fig), respectively. In addition, ortholog prediction and phylogenetic analysis of predicted *peritrophin-44 like* (ASTEI09413) showed a *Plasmodium* inhibition phenotype (summarized in S4C and S4D Fig). The neighbor-joining tree for all the selected genes was subjected to numerical re-sampling by bootstrapping, and the resulting bootstrap values were depicted at the tree branch nodes. Each value indicates the number of times (out of 1000 replicates) that the identified orthologs occurred in the re-samplings.

## Discussion

The intracellular bacterium *Wolbachia* has an extensively documented ability to block the infection of a number of insect hosts with various viruses and parasites, including several *Drosophila* viruses, the *Aedes*-vectored dengue and chikungunya viruses, and filarial nematodes, as well as *Plasmodium* in *Anopheles* mosquitoes [32–36]. The means by which *Wolbachia* blocks insect-borne pathogens are likely to rely on multiple mechanisms and to represent a diversity of insect-pathogen infection models [37–40]. Here we have investigated the basis of *Wolbachia* based anti-*Plasmodium* defense mechanisms in the *An*. *stephensi* mosquito malaria vector. We compared midgut and carcass transcriptomes between *Wolbachia*-infected and uninfected *An*. *stephensi* with and without blood feeding and found that *Wolbachia* infection significantly increased the expression of numerous immune genes in blood-fed midguts and carcasses and that some of the *Wolbachia*-modulated genes showed significant anti-*Plasmodium* activity. Our results provide insights into how *Wolbachia*-modulated immunity regulates infection with the human malaria parasite in *An*. *stephensi*.

Previous studies have demonstrated a major role for mosquito immunity in the defense against *Plasmodium* infection [41–43], and *Plasmodium* infection has been shown to upregulate the expression of a variety of mosquito immune genes [44]; apparently, anti-*Plasmodium* defenses are significantly regulated at the transcriptional level. Given the significant upregulation of numerous immune genes in *Wolbachia*-infected *An*. *stephensi*, we selected 18 of them in order to evaluate their potential role in defending against *Plasmodium* infection. Serine proteases (*CLIPB2* and *CLIPB4*), *TEP*s, *lysozyme C2* (*lysC2*), *peptidoglycan recognition protein 1 like* (*PGRP-LD*) and two novel genes, namely *peritrophin-44 like* and *macro domain*, were chosen because they showed significantly higher expression in both *Wolbachia*-infected *An*. *stephensi* and blood-fed midguts; these genes were also linked to mosquito immune defense against parasitic infection and infection with other pathogens [15,45,46].

Two CLIPs (CLIPB2 and CLIPB4) showed significantly higher mRNA abundance in *Wolbachia* infected blood-fed midgut and carcass tissues as well as in *Wolbachia*-uninfected *P*. *falciparum* infected *An. stephensi (Figs 4B and 4F and 5B).* Interestingly, the silencing of *CLIPB2* and *CLIPB4* significantly increased the oocyst loads and infection prevalence in the mosquito midguts, suggesting that these factors are *P*. *falciparum* antagonists that mediate *Wolbachia*-based suppression of *Plasmodium*. CLIP-domain serine proteases are a diverse group of proteolytic enzymes that are frequently involved in immune response signaling and amplification cascades (i.e., such as the Toll and PPO pathways) as well as developmental pathways (I.e., the Toll pathway) [47–49]. Studies of *An*. *gambiae* CLIPs have shown that the *CLIP-B* and *-C* families, including *CLIPB4*, *CLIPB8*, *CLIPB9*, *CLIPB10*, *CLIPB14*, *CLIPB17*, and *CLIPC9*, are upregulated during the parasite’s traversal of the midgut, and that they participate in the anti-*Plasmodium* defense by regulating *P*. *berghei* ookinete melanization [50–52]. Wang et al. (2021) have reported that silencing of *CLIPB15* leads to a significant decrease in *Phenoloxidase* (PO) activity, which acts as a catalyst for the formation of active intermediates of quinone for the synthesis of melanin, but silencing of *CLIPB22* does not alter the PO activity [48]. Silencing of both *CLIPB15* and *CLIPB22* affects the survival of *Ae*. *aegypti* after pathogenic bacterial infection [48]. Consistent with these studies, we identified three CLIP domain-containing serine proteases, two of which, *CLIPB2* and *CLIPB4*, were primarily upregulated in *Wolbachia*-infected blood-fed *An*. *stephensi* midguts and showed a significant anti-*Plasmodium* activity based on gene-silencing assays. Since CLIP domain serine proteases mediate anti-*Plasmodium* defenses as part of a complex network of regulatory cascades, it is impossible to predict all the effects of *Wolbachia*-induced altered expression of these genes. However, given the ability of serine proteases to act against *Plasmodium* infection, these CLIPs have the potential to be important for mosquitoes’ anti-*Plasmodium* immunity.

In addition, we observed upregulated expression of *lysozyme C2* in the *Wolbachia-*infected tissues and *Wolbachia-*uninfected *P*. *falciparu*m infected mosquitoes (Figs 4A and 5B). dsRNA-mediated knockdown of *lysozyme C2* resulted in a significant increase in *P*. *falciparum* infection intensity and prevalence when compared to those of the control GFP dsRNA-injected mosquitoes, suggesting it is a *Wolbachia*-inducible *P*. *falciparum* antagonist. Interestingly, the *An*. *gambiae lysozyme C-1* (*lysc1*) is a protective *Plasmodium* agonist [53] and has been shown to inhibit the melanization of non-biologic targets, thereby playing an opposite role to that of the *An*. *stephensi lysozyme C2* [54]. Studies have also shown that *An*. *gambiae lysC1* interacts directly with *Plasmodium* oocysts, and reducing *lysC1* lowers the parasite load in the mosquito host [53–55]. The increased oocyst load that we observed upon silencing of *lysozyme C2* (*lysC2*) suggests a possible interaction with oocysts. However, further investigation of a possible interaction between *lysC2* and *Plasmodium* will be required to understand the complexities of these intricate relationships. Based on the increased abundance of *PGRP-LD* (ASTEI02158) gene mRNAs in *Wolbachia-*infected tissues and *Wolbachia-*uninfected *P*. *falciparu*m infected mosquitoes (Figs 4G and 5B), we silenced *PGRP-LD* in *Wolbachia* uninfected *An*. *stephensi* mosquitoes followed by *P*. *falciparum* infection. Silencing of the predicted *PGRP-LD* (ASTEI02158) also led to an increased susceptibility to *P*. *falciparum* infection. This result is consistent with a previous study showing that *PGRP-LD* (ASTEI010245) maintains the homeostasis of the gut microbiota by negatively regulating innate immune responses and protecting the *An*. *stephensi* mosquitoes from malaria parasite infection [26]. These results further indicate that different isoforms of the *PGRP-LD* gene may exist in the *An*. *stephensi* genome.

Apart from *CLIP*s, *lysC*2, and *PGRP-LD*, we also selected three *TEP* genes whose expression levels were significantly higher in the *Wolbachia*-infected midgut tissue compared to the uninfected control. Significantly higher oocyst numbers were observed in *TEP4*- and *TEP15*- silenced mosquitoes compared to the control GFP dsRNA injected mosquitoes. However, the silencing of *TEP 14* (ASTEI0664) did not produce a phenotype similar to that observed for the other two *TEP* genes. A previous study has shown that transient wMelPop somatic infection in *An*. *gambiae* induces *TEP1* expression, and *Wolbachia*-induced *TEP1* upregulation contributes to the *Plasmodium* suppression [6].

From the pool of *Wolbachia*-regulated *An*. *stephensi* genes, we also selected six genes with no predicted immunity or anti-*Plasmodium* functions; *peritrophin-44-like*, *DUF4794 domain*, *macro domain*, *transcription factor MafK*, *carboxypeptidase A*, and *CAP10 domain*. We assessed their potential role in modulating *Plasmodium* infection using our standard gene-silencing and infection assays. These genes showed increased expression in *Wolbachia* LB1-infected blood-fed midgut tissues and in *P*. *falciparum*-infected *An*. *stephensi*. RNAi-mediated knockdown of the *DUF4794 domain*, *transcription factor MafK*, *carboxypeptidase A*, *and CAP10 domain* genes had no effect on *Plasmodium* infection; however, knockdown of *peritrophin-44-like* and *macro domain* -encoding gene led to a significant increase in the number of oocysts per midgut. While the oocyst counts were increased after knockdown of either macro domain or peritrophin-44, only silencing of the peritrophin-44-like protein gene had a statistically significant effect on infection prevalence. *Macro domain-containing proteins* (ASTEI09413) are not well characterized in insects [56,57]; therefore, an association with the *Wolbachia*-induced anti-*Plasmodium* defense could be an interesting new field to explore. Similarly, little information is available about the *peritrophin-44-like gene* in insect–parasite interactions. Elvin *et al*. (1996) characterized peritrophin-44 in the fly *Lucilia cuprina* and suggested that it plays important roles in the maintenance of insect gut structure, facilitation of digestion, and protection of digestive epithelial cells from bacterial damage and parasitic invasion [58]. Additionally, among the 18 selected genes, those encoding the predicted *CLIP-like*, *3-glucan binding protein*, *DUF4794 domain*, *transcription factor MafK*, *CAP10 domain*, *carboxypeptidase A*, and *TEP14* did not show any influence on *P*. *falciparum* infection upon gene-silencing (Figs 4 and 6).

Altogether, our study demonstrates that *Wolbachia*-regulated immune genes such as *CLIP* domain serine proteases, *lysozyme C2*, *PGRP-LD*, and *TEP*s have a significant inhibitory or antagonistic effect on *Plasmodium* development and are therefore likely to mediate *Wolbachia*’s suppression of *P*. *falciparum* infection. Also, we show here that two novel genes, macro domain containing protein and peritrophin-44, also play a likely role in *Wolbachia*-mediated *Plasmodium* suppression. We did not perform gene expression or mRNA silencing assays with co-infections (*Wolbachia* and *Plasmodium*) due to difficulties with data interpretation when tripartite (*Wolbachia*- *Anopheles*- *Plasmodium*) interactions are addressed. Silencing of a *Wolbachia* induced gene in a coinfection assay could for example either affect *Plasmodium* directly, or indirectly through a change in *Wolbachia* infection. To our knowledge, this is the first-time links between these genes and the *Wolbachia* infection-modulated *Plasmodium* suppression has been established in a stably *Wolbachia-*transinfected mosquito and therefore warrants further investigation. Furthermore, we assessed effects of *Wolbachia* infection on the early midgut stages that will be influenced by *Wolbachia*-regulated gene expression occurring prior to *Plasmodium* introduction to the mosquito midgut. A greater understanding of these interactions may facilitate the development of future *Wolbachia*-based malaria control strategies as has been done for dengue control.

## Material and methods

### Ethics statement

This study was conducted in accordance with the recommendations in the Guide for the Care and Use of Laboratory Animals of the National Institutes of Health, the Animal Care and Use Committee (ACUC) of Johns Hopkins University, and the institutional Ethics Committee. The Institutional Animal Care and Use Committee (IACUC) approved the protocol RA21H388. Mice were used for mosquitoes rearing. Anonymous, commercial blood from human donors was used for *Plasmodium falciparum* gametocyte cultures and infection assays in mosquitoes.

### Mosquito rearing

The wild-type *An*. *stephensi* LIS strain (*Wolbachia*-free) and *An*. *stephensi* LB1 strain (*w*AlbB-infected) were reared as described previously [15]. For RNAseq experiments, O+ whole blood with citrate-phosphate dextrose (CPD) as anticoagulant, was used for blood-feeding mosquitoes.

### RNA extraction and real-time PCR

Total RNA was extracted from the pool of ten laboratory-reared sugar- and blood-fed *An*. *stephensi* mosquitoes using TRIzol (Invitrogen, USA) followed by Turbo Dnase I treatment. Similarly, midguts and carcass tissues were dissected in 1X PBS from *Wolbachia*-infected (wAlbB or LB1) or uninfected 5- to 6-day-old female LIS mosquitoes and collected in TRIzol reagent for RNA extraction. Complementary DNA (cDNA) was synthesized using 1 μg of total RNA with oligo dT primers and Moloney murine leukemia virus (MMLV) reverse transcriptase (Promega, USA). Real-time PCR was done using SYBR green PCR Master Mix (Applied Biosystem) with a final volume of 15 μl in a StepOne real-time PCR system (Applied Biosystem). For all assays, the expression of selected genes was normalized to the expression of the ribosomal protein S7 gene (Gene ID-MF999156.1). Three replicates were used per gene and, for tissue-specific expression, samples with five females were pooled to make one replicate. The relative expression of selected genes was calculated as a 2^-ΔΔCT^ value between sugar-fed and blood-fed, *P*. *falciparum*-infected and uninfected, and *Wolbachia*-infected (LB1) and uninfected *An*. *stephensi*. Specifically, we normalized the expression reads of blood-fed with sugar fed and *P*. *falciparum* infected with uninfected. Similarly, *Wolbachia* uninfected was used as normalizing control for assessing the expression of each gene in *Wolbachia* infected tissues. The sequences of all primers are given in S3 Table.

### RNA sequencing and bioinformatics analysis

Total RNA was extracted from midguts and carcasses of *Wolbachia*-infected (LB1) and uninfected (LIS) one-week-old female mosquitoes using TRIzol. The quality of the RNA was assessed by Agilent 2100, and mRNA was enriched using oligo (dT) beads. An Illumina sequencing library was constructed for each RNA sample according to the manufacturer’s instructions and sequenced by Novogene Co., LTD (Beijing, China) on the Illumina platform with paired-end 150 bp (PE 150). Data was deposited in NCBI’s Sequence Read Archive (SRA: SUB14206326). Raw data in FASTQ format were processed to remove reads containing adapters, reads containing ploy-N, and low-quality reads; clean reads were aligned to the *An*. *stephensi* genome (VectorBase-57). Feature Counts was used to quantify transcript abundance in each sample by using the gene annotation obtained from VectorBase. Differentially expressed (DE) genes between LB1 and LIS mosquitoes in midguts and carcasses were identified with DESeq2. Gene ontology (GO) and -fold enrichment of the DE genes was analyzed using the built-in program in Vectorbase or ShinyGO (http://bioinformatics.sdstate.edu/go/). The interaction networks of the DE genes were generated with Revigo (http://revigo.irb.hr/) and modified by Cytoscape [59].

### dsRNA synthesis and RNAi-mediated gene silencing

PCR fragments of 600–700 bp were amplified with each selected gene-specific primer, tailed with a short T7 promoter sequence, 5’taatacgactcactataggg’3, using the cDNA from sugar-fed *An. stephensi* female mosquitoes as template. Each PCR fragment was purified, and specific dsRNA was synthesized using the HiScribeT7 Quick HighYield RNA synthesis kit (New England Biolabs) according to the manufacturer’s protocol. The PCR fragment for GFP that served as a control was amplified using a plasmid template containing the GFP gene [60]. The concentration and quality of the dsRNA were determined spectrophotometrically by Nanodrop1000 (company) and agarose gel electrophoresis. The gene-specific primers used for dsRNA synthesis are summarized in S3 Table. For nano-injections, three-day-old *An*. *stephensi* Lis female mosquitoes were cold-anesthetized and injected intrathoracically with 69 nl of a 3μg/ul dsRNA for each gene of interest. A control group of mosquitoes were injected with dsGFP. All injections were repeated 3–5 times using a Nanoject microinjector, and approximately 80–100 mosquitoes were silenced for each gene per experiment. Each biological replicate corresponds to a different mosquito population cage. After dsRNA injection, mosquitoes were left for 3 days under optimal insectary conditions, with 10% sucrose as the sugar source [23,61].

### Gene silencing efficiency

The efficiency of gene silencing was determined 3 days post-dsRNA injection by real-time quantitative reverse transcription-PCR (qRT-PCR) for all the selected genes, along with control dsGFP. Total RNA was extracted from a pool of five to six mosquitoes, followed by cDNA synthesis as described in the previous section. qPCR was performed using the respective gene primers in a StepOnePlus real-time PCR machine as mentioned previously. The primer sequences used for silencing validation are given in S3 Table.

### *Plasmodium falciparum* infection

To determine the anti-*Plasmodium* activity, gene-knockdown female mosquitoes were starved for 4–5 h. In brief, 14-to 16-day-old *P*. *falciparum* NF54 cultures were diluted to 0.1–0.3% with fresh red blood cells (RBCs), and 60% human serum was added to the final volume of infected blood. Starved female mosquitoes were allowed to feed on the infected blood through artificial membrane feeders maintained at 37°C for 30–45 min [23,61]. After feeding, the unfed females were removed, and the fed mosquitoes were kept for 8–10 days at 27°C for oocyst counting. Midguts were dissected on the 8^th^ day post-feeding in phosphate-buffered saline (PBS) and stained with 0.2% mercurochrome to determine the oocyst load under a light microscope.

### Phylogenetic analysis

The full-length sequences of selected genes and their putative orthologs in *An*. *gambiae* were retrieved from VectorBase and aligned in the FASTA format using the Mafft multiple sequence alignment tool. Each amino acid residue was aligned pairwise and compared with other residues of the same row, and identical residues between the species were marked with colored residues based on their biochemical properties. Phylogenetic relationships between the *An*. *stephens*i selected gene sequences and their respective orthologs from *An*. *gambiae* and *An*. *coluzzii* were analyzed using MEGA 11.0.13 [62]. Phylogenetic trees were constructed by neighbor-joining method. Protein sequences of the target genes of *An*. *stephensi* were aligned with their corresponding putative orthologs from *An*. *gambiae* and *An*. *coluzzii*, and phylogenetic analysis was performed.

### Statistical analysis

For *Plasmodium* infection experiments, the dot plots for oocysts/midgut were generated with GraphPad Prism5 software. Statistical differences between three independent biological replicates were assessed, and the data were pooled. The significance of the differences in infection load (the number of oocysts per midgut) and prevalence (the number of infected mosquitoes per total number of mosquitoes examined) between the control dsGFP and the gene-silenced groups were determined through nonparametric Mann-Whitney tests and the Fisher’s exact test, respectively. Two-tailed P values are given for all experiments in the figure legends along with information concerning the total number of midguts, median, and infection prevalence. Data from three biological replicates for each experiment were shown in all figures.

## Supporting information

S1 Fig Relative transcript levels of various selected genes in wild *An*. *stephensi* at 3 days post-dsRNA injection with the respective genes or with dsGFP as a control. The data are presented as means± SD of three biological replicates.
(TIF)

S2 Fig Evolutionary relationships of *An*. *stephensi* ASTEI08922, ASTEI08922, and ASTEI02516 with *An*. *gambiae* CLIPs. (**A**) Amino acid pairwise alignment of full-length *An*. *stephensi* unspecified genes and their putative orthologs in *An*. *gambiae*. (**B**) Phylogenetic tree (neighbor-joining) of *An*. *stephensi* unspecified genes and their putative *An*. *gambiae* orthologs. Based on bootstrap values and clustering, ASETI08922 and ASTEI08923 were predicted to be CLIPB4 and CLIPB2, respectively. (**C**) Amino acid pairwise alignment of the full-length *An*. *stephensi* unspecified gene ASTEI02158 and its putative orthologs in *An*. *gambiae*.
(TIF)

S3 Fig Evolutionary relationships of *An*. *stephensi* ASTEI01308 with *An*. *gambiae*. (**A**) Amino acid pairwise alignment of the full-length *An*. *stephensi* unspecified product and its putative orthologs in *An*. *gambiae*. (**B)** Phylogenetic tree (neighbor-joining) of *An*. *stephensi* unspecified product and its putative *An*. *gambiae* orthologs. Based on bootstrap values and clustering, gene ID ASETI01308 is predicted to be *lysC2*-like.
(TIF)

S4 Fig Evolutionary relationships of *An*. *stephensi* ASTEI06644, ASTEI08428, and ASTEI06643 with *An*. *gambiae* TEPs. (**A**) Amino acid pairwise alignment of the *An*. *stephensi* unspecified genes and their putative orthologs in *An*. *gambiae*. (**B**) Phylogenetic tree (neighbor-joining) of *An*. *stephensi* unspecified genes ASTEI06644, ASTEI08428, and ASTEI06643 and their putative *An*. *gambiae* orthologs. (**C)** and (**D**) Amino acid pairwise alignment and phylogenetic tree of the full-length *An*. *stephensi* unspecified gene ASTEI09413 and its putative orthologs in *An*. *coluzzii*. Bootstrap values were presented at the tree branch nodes.
(TIF)

S1 Table Quantitative control and alignment summary of transcriptome data. (XLSX)

S2 Table *Wolbachia*-regulated immune genes in midguts and carcasses of sugar-fed and blood-fed *An*. *stephensi*. (XLSX)

S3 Table Primers used in this study. (XLSX)
