# Supplementary figures and images for: Wolbachia infection-responsive immune genes suppress Plasmodium falciparum infection in Anopheles stephensi
Source: PLoS Pathog. 2024 Apr 10;20(4):e1012145. doi: 10.1371/journal.ppat.1012145 (PMC11034644; doi:10.1371/journal.ppat.1012145)

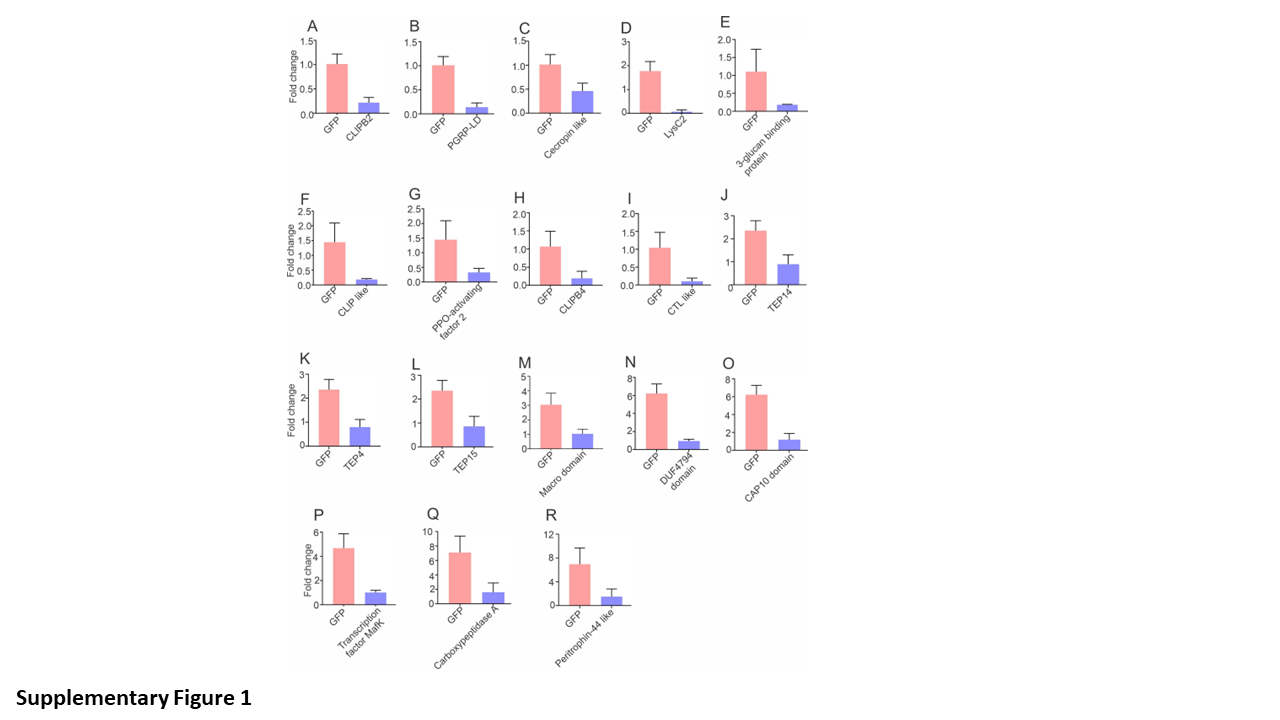

Supplement: S1 Fig — The data are presented as means± SD of three biological replicates. (TIF) [file ppat.1012145.s001.TIF]

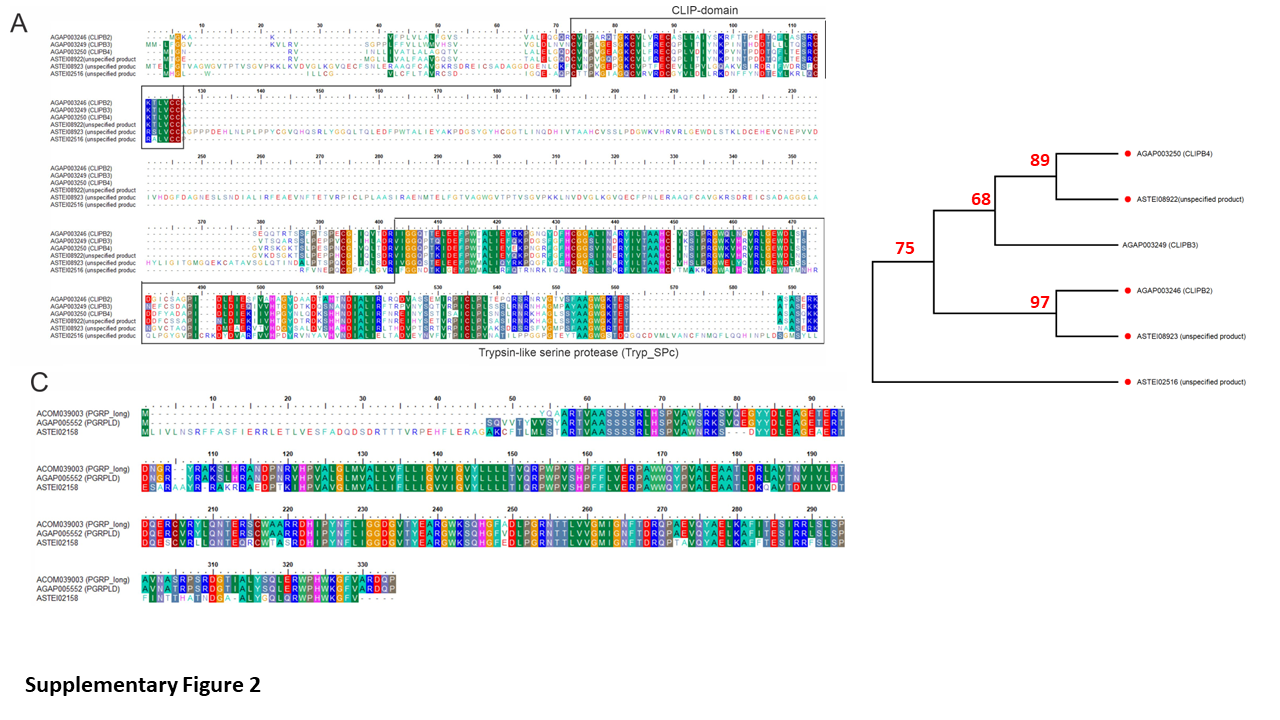

Supplement: S2 Fig — (A) Amino acid pairwise alignment of full-length An. stephensi unspecified genes and their putative orthologs in An. gambiae. (B) Phylogenetic tree (neighbor-joining) of An. stephensi unspecified genes and their putative An. gambiae orthologs. Based on bootstrap values and clustering, ASETI08922 and ASTEI08923 were predicted to be CLIPB4 and CLIPB2, respectively. (C) Amino acid pairwise alignment of the full-length An. stephensi unspecified gene ASTEI02158 and its putative orthologs in An. gambiae. (TIF) [file ppat.1012145.s002.TIF]

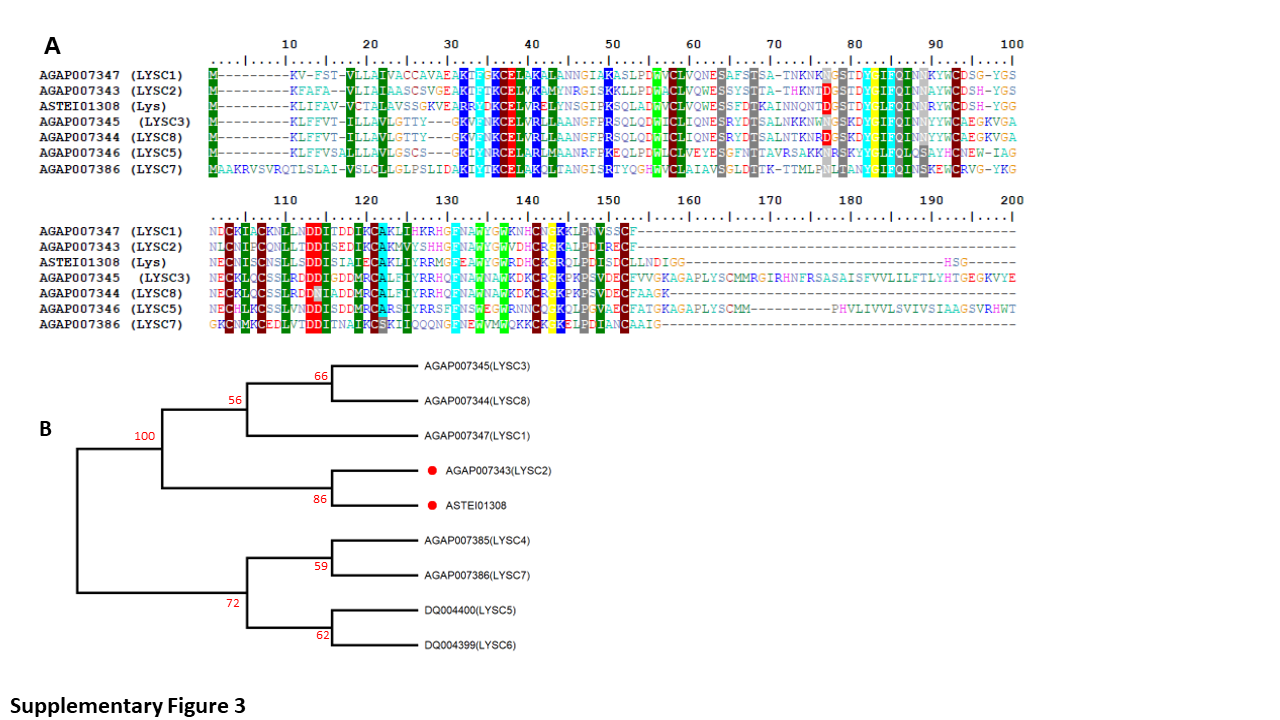

Supplement: S3 Fig — (A) Amino acid pairwise alignment of the full-length An. stephensi unspecified product and its putative orthologs in An. gambiae. (B) Phylogenetic tree (neighbor-joining) of An. stephensi unspecified product and its putative An. gambiae orthologs. Based on bootstrap values and clustering, gene ID ASETI01308 is predicted to be lysC2-like. (TIF) [file ppat.1012145.s003.TIF]

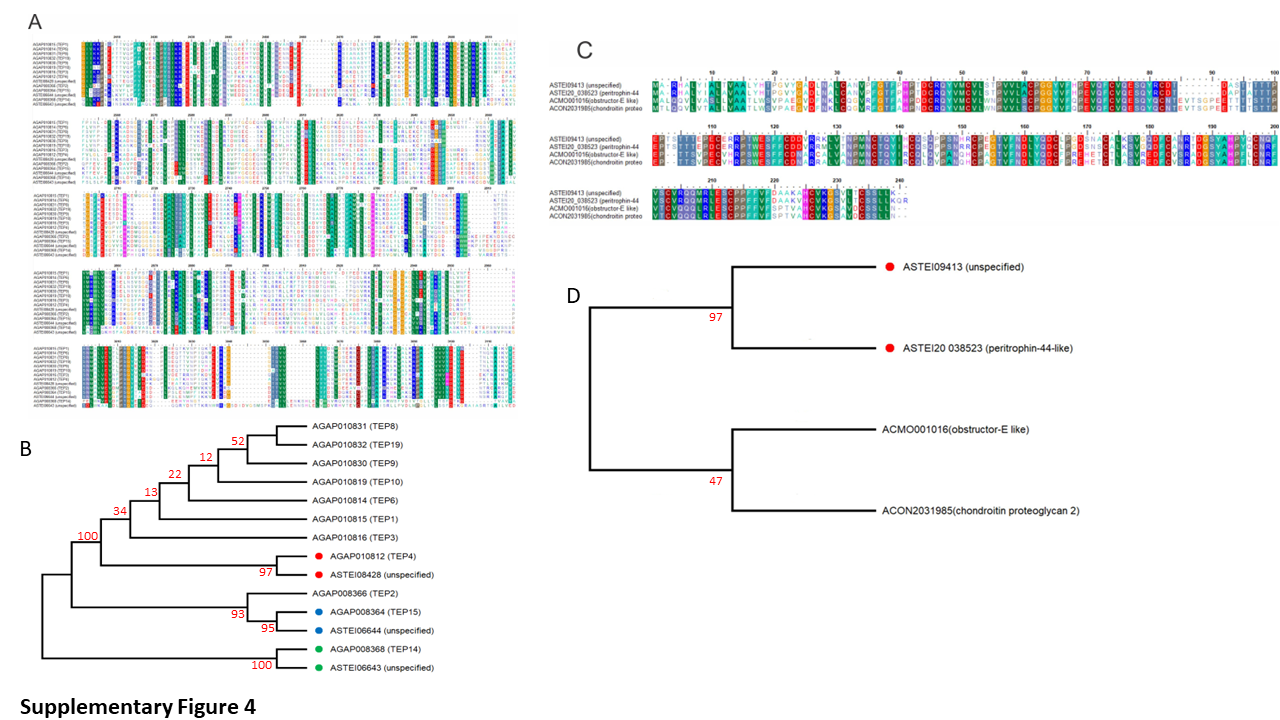

Supplement: S4 Fig — (A) Amino acid pairwise alignment of the An. stephensi unspecified genes and their putative orthologs in An. gambiae. (B) Phylogenetic tree (neighbor-joining) of An. stephensi unspecified genes ASTEI06644, ASTEI08428, and ASTEI06643 and their putative An. gambiae orthologs. (C) and (D) Amino acid pairwise alignment and phylogenetic tree of the full-length An. stephensi unspecified gene ASTEI09413 and its putative orthologs in An. coluzzii. Bootstrap values were presented at the tree branch nodes. (TIF) [file ppat.1012145.s004.TIF]
